# FeO_x_-Modified Ultrafine Platinum Particles Supported on MgFe_2_O_4_ with High Catalytic Activity and Promising Stability toward Low-Temperature Oxidation of CO

**DOI:** 10.3390/molecules29051027

**Published:** 2024-02-27

**Authors:** Chanchan Wang, Fen Wang, Jianjun Shi

**Affiliations:** 1 School of Chemical and Blasting Engineering, Anhui University of Science and Technology, Huainan 232001, China; chanchanwang08@163.com; 2 Institute of Environment-Friendly Materials and Occupational Health of Anhui University of Science and Technology (Wuhu), Wuhu 241003, China

**Keywords:** catalyst, ultrafine platinum particles, MgFe_2_O_4_, FeO_x_ modification, CO oxidation

## Abstract

Catalytic oxidation is widely recognized as a highly effective approach for eliminating highly toxic CO. The current challenge lies in designing catalysts that possess exceptional low-temperature activity and stability. In this work, we have prepared ultrafine platinum particles of ~1 nm diameter dispersed on a MgFe_2_O_4_ support and found that the addition of 3 wt.% FeO_x_ into the 3Pt/MgFe_2_O_4_ significantly improves its activity and stability. At an ultra-low temperature of 30 °C, the CO can be totally converted to CO_2_ over 3FeO_x_-3Pt/MgFe_2_O_4_. High and stable performances of CO-catalytic oxidation can be obtained at 60 °C on 3FeO_x_-3Pt/MgFe_2_O_4_ over 35 min on-stream at WHSV = 30,000 mL/(g·h). Based on a series of characterizations including BET, XRD, ICP, STEM, H_2_-TPR, XPS, CO-DRIFT, O_2_-TPD and CO-TPD, it was disclosed that the relatively high activity and stability of 3FeO_x_-3Pt/MgFe_2_O_4_ is due to the fact that the addition of FeO_x_ could facilitate the antioxidant capacity of Pt and oxygen mobility and increase the proportion of adsorbed oxygen species and the amounts of adsorbed CO. These results are helpful in designing Pt-based catalysts exhibiting higher activity and stability at low temperatures for the catalytic oxidation of CO.

## 1. Introduction

The incomplete combustion of carbon-containing compounds and some important industrial processes such as ironmaking and steelmaking, as well as vehicle exhaust, produce carbon monoxide (CO) widely [1,2]. CO is a highly toxic gas for humans, and exposure to ppm levels of CO in an enclosed environment can cause serious poisoning. In industry, the trace amounts of CO can poison the Fe-based ammonia-synthesis catalysts [3], Pt-based fuel cell [4] and hydrogenation [5] catalysts, and so on. Catalytic oxidation is recognized as a highly effective method for removing low concentrations of CO. The major challenge of CO catalytic oxidation is to design catalysts with outstanding performance at low temperatures. 

The catalysts employed in CO oxidation mainly consisted of transition-metal oxides (such as CuO [6], MnO_2_ [7] and Co_3_O_4_ [8]) and noble metal catalysts (like supported Au [9], Pt [10], Pd [11], and Rh [12]). The attractive activity and thermal stability of noble metal-based catalysts make them highly appealing for CO oxidation compared to transition-metal oxide catalysts. Among numerous reported noble metal-based catalysts, Pt-based catalysts have gained great interest in various catalytic oxidation reactions, including formaldehyde oxidation [13], CO oxidation [14], propane oxidation [15], and so on. It is well known that ultrafine platinum particles (<2 nm) can efficiently enhance the catalytic oxidation activity [16,17], probably owing to the increase in their surface area and the presence of more edge and corner atoms. In addition, decreasing the size of platinum particles can significantly improve the Pt atom utilization efficiency, and therefore can save catalyst costs. The typical methods of preparing ultrafine metallic platinum particles are colloidal synthesis. However, the usage of organic capping agents during the colloidal synthesis process often blocks the catalytic active sites, thereby causing a decrease in catalytic activity. Therefore, using the capping agents-free synthesis method to prepare ultrafine metallic platinum particles is more advantageous. It was reported that FeO_x_ possesses an excellent ability to disperse Pt owing to the strong interaction of Pt–FeO_x_ species [18]. Considering the fact that the single metal oxide has a lower surface area and poorer thermal stability than spinel materials, the usages of iron-based spinel oxides such as MgFe_2_O_4_ to disperse Pt are very promising. Additionally, it has been reported that the incorporation of FeO_x_ as an additive can boost the activities of supported Pt catalysts in the oxidation of CO [19,20,21]. Thus, we expected that FeO_x_ modification further improves the activities of MgFe_2_O_4_-supported Pt catalysts. 

In this paper, we developed FeO_x_-modified MgFe_2_O_4_-supported Pt catalysts with ultra-small-sized Pt particles of ~1 nm. It was found that FeO_x_ modification significantly promotes the activity and stability of Pt/MgFe_2_O_4_ in the oxidation of CO reactions, and the CO conversion can reach 100.0% at the low temperature of 30 °C. The as-prepared catalyst structure was comprehensively characterized by using BET, ICP, XRD, STEM, H_2_-TPR, XPS, CO-DRIFT, O_2_-TPD, and CO-TPD. The results reveal that the interaction between FeO_x_ and Pt exists, and the transfer of electrons from Pt to FeO_x_ was determined. The incorporation of 3 wt.% FeO_x_ into the 3Pt/MgFe_2_O_4_ improved the antioxidant capacity of Pt, increased the ratio of adsorbed oxygen species and the adsorbed amount of CO and promoted oxygen mobility, which are regarded as the main reasons responsible for the higher performance for CO oxidation on 3FeO_x_-3Pt/MgFe_2_O_4_.

## 2. Results

### 2.1. Catalyst Performance

**Figure 1:**
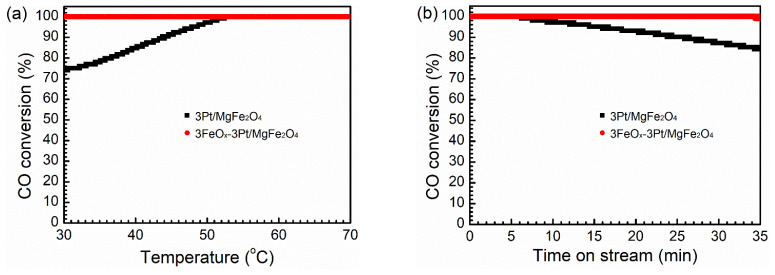
(**a**) CO catalytic-oxidation activities and (**b**) catalytic stability at 60 °C of 3Pt/MgFe_2_O_4_ and 3FeO_x_-3Pt/MgFe_2_O_4_ at WHSV = 30,000 mL/(g·h).

Figure 1a displays the CO oxidation activities of 3Pt/MgFe_2_O_4_ and 3FeO_x_-3Pt/MgFe_2_O_4_ at 30–70 °C at WHSV = 30,000 mL/(g·h). The conversion of CO over 3Pt/MgFe_2_O_4_ displayed an increase from 74.0% to 100.0% as the reaction temperature was elevated from 30 to 52 °C. However, the 100.0% conversion of CO was achieved within the range of 30–70 °C over 3FeO_x_-3Pt/MgFe_2_O_4_. Figure 1b displays the catalytic stability of the 3Pt/MgFe_2_O_4_ and 3FeO_x_-3Pt/MgFe_2_O_4_ catalysts in the CO oxidation reaction at 60 °C. It is obvious that the 3FeO_x_-3Pt/MgFe_2_O_4_ catalyst displays a stable CO conversion of 100.0% during the total test time of 35 min, while the CO conversion over 3Pt/MgFe_2_O_4_ decreases from 100.0% to 84.2% in the last 29 min of the reaction. Based on the results provided above, it can be inferred that the incorporation of 3 wt.% FeO_x_ into 3Pt/MgFe_2_O_4_ is beneficial in enhancing the CO-oxidation performance.

### 2.2. Catalyst Characterization

**Figure 2:**
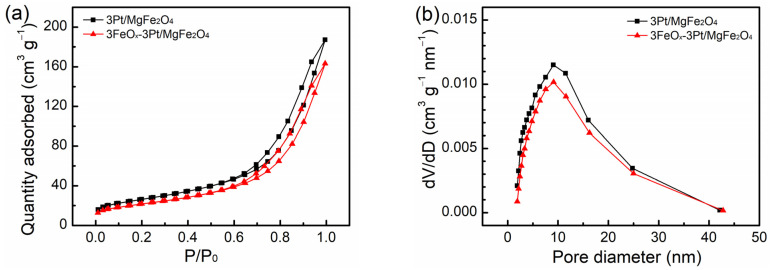
(**a**) Isotherms of nitrogen physical adsorption and (**b**) pore-size distribution plots for the fresh 3Pt/MgFe_2_O_4_ and 3FeO_x_-3Pt/MgFe_2_O_4_.

The isotherms of nitrogen physical adsorption as well as the pore-size distribution curves for the fresh 3Pt/MgFe_2_O_4_ and 3FeO_x_-3Pt/MgFe_2_O_4_ samples are presented in Figure 2. Both 3Pt/MgFe_2_O_4_ and 3FeO_x_-3Pt/MgFe_2_O_4_ samples demonstrated type IV isotherms accompanied by hysteresis loops, which are indicative of their mesoporous structure (Figure 2a). The pore-size distributions plot also showed a mesoporous structure for both samples (Figure 2b). The corresponding properties of the 3Pt/MgFe_2_O_4_ and 3FeO_x_-3Pt/MgFe_2_O_4_ are listed in Table 1. Based on the nitrogen-adsorption results, the specific surface areas for the 3Pt/MgFe_2_O_4_ and 3FeO_x_-3Pt/MgFe_2_O_4_ samples are 93.8 and 77.9 m^2^/g, respectively. Additionally, the average pore sizes were determined to be 11.3 and 11.5 nm, and the pore volumes were measured to be 0.29 and 0.25 cm^3^/g for the 3Pt/MgFe_2_O_4_ and 3FeO_x_-3Pt/MgFe_2_O_4_ samples, respectively. The Pt loadings, as determined by ICP-OES, are 3.1 and 2.9 wt.% for the fresh 3Pt/MgFe_2_O_4_ and 3FeO_x_-3Pt/MgFe_2_O_4_, respectively.

**Figure 3:**
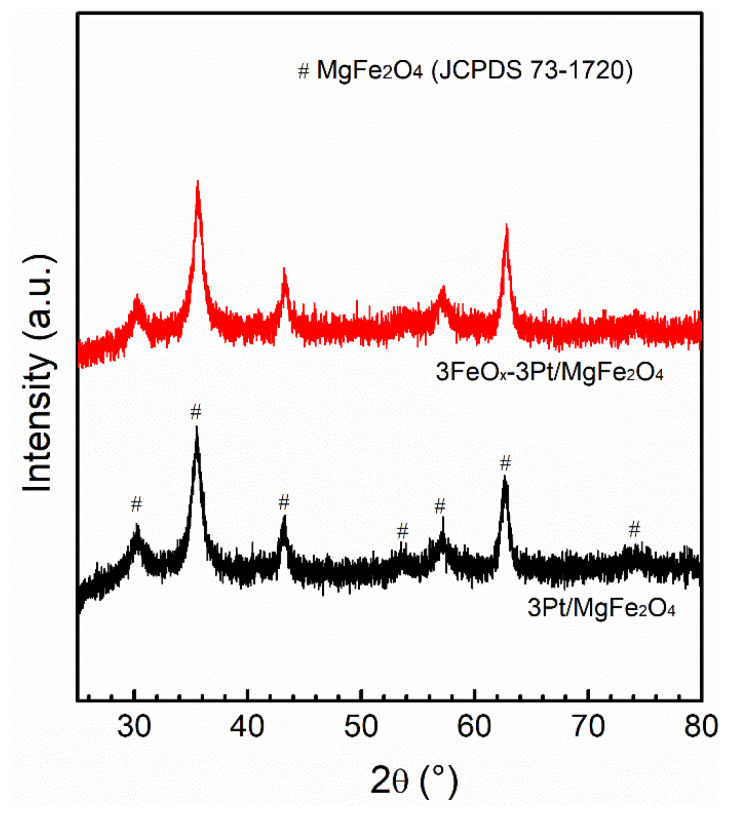
XRD patterns of the fresh 3Pt/MgFe_2_O_4_ and 3FeO_x_-3Pt/MgFe_2_O_4_.

**Figure 4:**
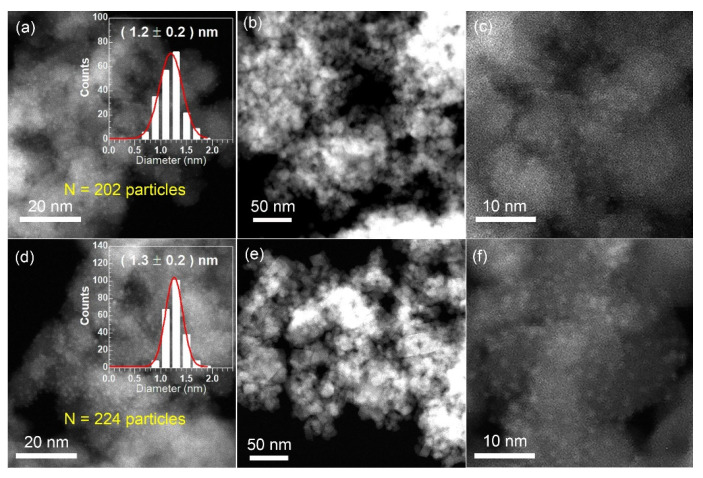
STEM images and the size histogram of Pt-particle distribution for the fresh (**a**–**c**) 3Pt/MgFe_2_O_4_ and (**d**–**f**) 3FeO_x_-3Pt/MgFe_2_O_4_.

The XRD patterns for the fresh 3Pt/MgFe_2_O_4_ and 3FeO_x_-3Pt/MgFe_2_O_4_ are presented in Figure 3. The characteristic diffraction peaks for MgFe_2_O_4_ (JCPDS 73-1720) can be observed in both 3Pt/MgFe_2_O_4_ and 3FeO_x_-3Pt/MgFe_2_O_4_. In addition to MgFe_2_O_4_, no diffraction peak of Pt was observed on these two catalysts, suggesting that the sizes of Pt nanoparticles are smaller than that of the XRD detection limit (<3 nm). To further confirm this result, the STEM technique was employed. As shown in Figure 4a,d, both the fresh 3Pt/MgFe_2_O_4_ and 3FeO_x_-3Pt/MgFe_2_O_4_ have high Pt-nanoparticle density with narrow size distributions. According to statistical data of over 200 Pt-nanoparticle sizes, the average sizes of Pt nanoparticles were determined to be 1.2 and 1.3 nm for 3Pt/MgFe_2_O_4_ and 3FeO_x_-3Pt/MgFe_2_O_4_, respectively. Pt nanoparticles in 3Pt/MgFe_2_O_4_ and 3FeO_x_-3Pt/MgFe_2_O_4_ can be seen more clearly in Figure 4c,f.

**Figure 5:**
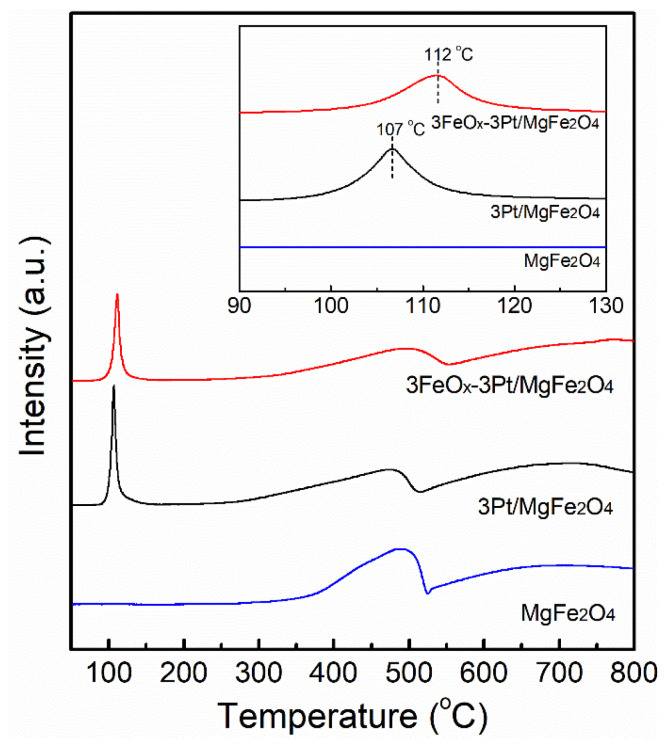
H_2_-TPR diagrams of the calcined 3Pt/MgFe_2_O_4_ and 3FeO_x_-3Pt/MgFe_2_O_4_ as well as MgFe_2_O_4_ support.

We conducted H_2_-TPR experiments to explore in detail the reducibility of 3Pt/MgFe_2_O_4_ and 3FeO_x_-3Pt/MgFe_2_O_4_ as well as MgFe_2_O_4_ support, and the obtained outcomes are illustrated in Figure 5. For MgFe_2_O_4_ support, the reduction peak above 250 °C belongs to the reduction of iron species because of the nonreducible of Mg^2+^. For 3Pt/MgFe_2_O_4_ and 3FeO_x_-3Pt/MgFe_2_O_4_, a distinct reduction peak was found at 90–150 °C in addition to a reduction peak above 250 °C, which belongs to the PtO_x_ reduction [22,23]. As shown in insert figure, the PtO_x_ reduction peak for 3Pt/MgFe_2_O_4_ and 3FeO_x_-3Pt/MgFe_2_O_4_ is centered at 107 and 112 °C, respectively. The introduction of FeO_x_ to 3Pt/MgFe_2_O_4_ raises the reduction temperature of PtO_x_ species obviously. It suggests that the interaction that exists between FeO_x_ and PtO_x_ and FeO_x_ is expected to modify the electron properties of metallic Pt nanoparticles.

**Figure 6:**
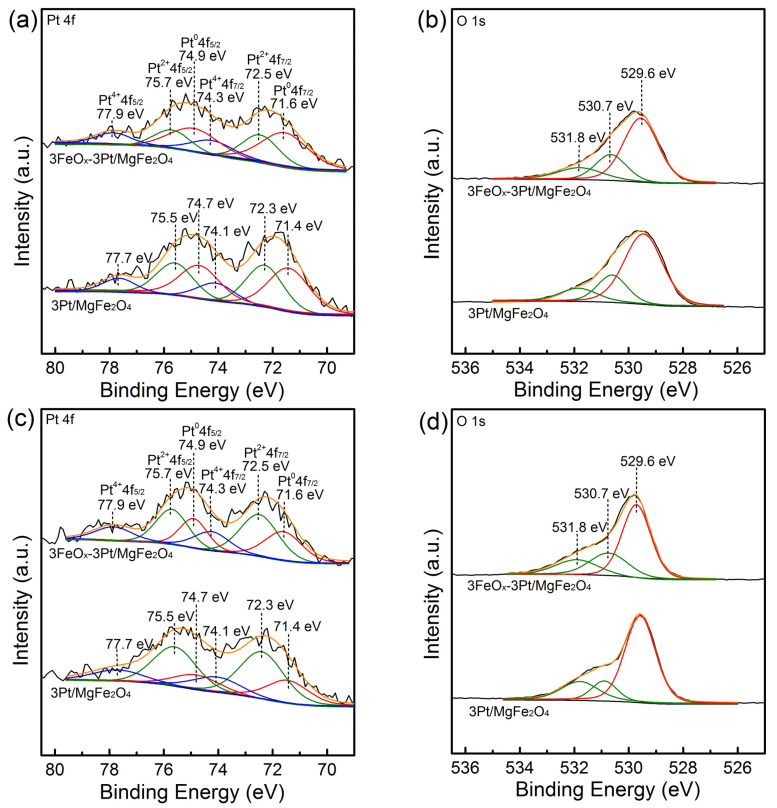
(**a**,**c**) Pt 4f and (**b**,**d**) O 1s XPS spectra for the (**a**,**b**) fresh and (**c**,**d**) spent 3Pt/MgFe_2_O_4_ and 3FeO_x_-3Pt/MgFe_2_O_4_.

The valence states of platinum and the type of surface oxygen species in 3Pt/MgFe_2_O_4_ and 3FeO_x_-3Pt/MgFe_2_O_4_ were explored using XPS. The XPS spectra for the Pt 4f orbitals of the fresh 3Pt/MgFe_2_O_4_ and 3FeO_x_-3Pt/MgFe_2_O_4_ are presented in Figure 6a. These spectra can be fitted well with six peaks. The Pt 4f spectra of the fresh 3Pt/MgFe_2_O_4_ exhibit two fitted peaks observed at binding energy values of 71.4 and 74.7 eV, which can be ascribed to Pt 4f_7/2_ and Pt 4f_5/2_ orbitals for Pt^0^, respectively. Two fitted peaks observed at binding energy values of 72.3 and 75.5 eV can be associated with Pt 4f_7/2_ and Pt 4f_5/2_ orbitals for Pt^2+^, respectively. The presence of two additional peaks observed at 74.1 and 77.7 eV can be assigned to Pt 4f_7/2_ and Pt 4f_5/2_ orbitals of the Pt^4+^ state, respectively [24,25,26,27]. Similar Pt species were observed in the fresh 3FeO_x_-3Pt/MgFe_2_O_4_ while their binding energy shifted to higher values by ~0.2 eV. This suggests that there is an interaction between Pt and FeO_x_ where the electrons are transferred from Pt to FeO_x_. The existence of Pt^2+^ and Pt^4+^ in the fresh 3Pt/MgFe_2_O_4_ and 3FeO_x_-3Pt/MgFe_2_O_4_ samples could be induced by the oxidation process with air at room temperature. According to the calculation of the proportion of the Pt^0^ peak areas to the sum of the Pt^0^, Pt^2+^ and Pt^4+^ peak areas, the relative amounts of Pt^0^ in the fresh 3Pt/MgFe_2_O_4_ and 3FeO_x_-3Pt/MgFe_2_O_4_ are 43.1% and 49.0%, respectively (Table 1). These findings indicate that FeO_x_ reduced the degree of reoxidation for the reduced-state Pt, which indirectly reflects the Pt–FeO_x_ interaction in the 3FeO_x_-3Pt/MgFe_2_O_4_ sample. Figure 6b presents the XPS spectra for the O 1s orbitals of the fresh 3Pt/MgFe_2_O_4_ and 3FeO_x_-3Pt/MgFe_2_O_4_ samples. The spectra could be fitted well, with three peaks which are located at binding energy values of 529.6, 530.7 and 531.8 eV, respectively. The peak centered at the low binding energy of 529.6 eV corresponds to the lattice oxygen (O_latt_), where the peaks centered at the high binding energies of 530.7 and 531.8 eV correspond to adsorbed oxygen (O_ads_) species [28,29,30]. According to the proportion of the O_ads_ peak areas to the sum of the O_latt_ and O_ads_ peak areas, the respective proportions of O_ads_ in the fresh 3Pt/MgFe_2_O_4_ and 3FeO_x_-3Pt/MgFe_2_O_4_ are 31.7% and 37.4%, respectively. This indicates that the 3FeO_x_-3Pt/MgFe_2_O_4_ possesses a higher percentage of adsorbed oxygen species, indicating the higher capacity to adsorb and activate molecular oxygen, and therefore leading to enhanced catalyst performance [21,31,32]. Figure 6c,d present the Pt 4f and O 1s XPS results for the spent 3Pt/MgFe_2_O_4_ and 3FeO_x_-3Pt/MgFe_2_O_4_ catalysts. In the spent 3Pt/MgFe_2_O_4_ and 3FeO_x_-3Pt/MgFe_2_O_4_, the binding energies of Pt 4f and O 1s remained unchanged. The relative amounts of Pt^0^ for the spent 3Pt/MgFe_2_O_4_ and 3FeO_x_-3Pt/MgFe_2_O_4_ are 24.5% and 31.7%, respectively. The higher percentage of Pt^0^ in the spent 3FeO_x_-3Pt/MgFe_2_O_4_ suggests that FeO_x_ can improve the antioxidant performance of Pt/MgFe_2_O_4_ in the reaction atmosphere, which always leads to a high CO oxidation performance [25]. The relative amounts of O_ads_ for the spent 3Pt/MgFe_2_O_4_ and 3FeO_x_-3Pt/MgFe_2_O_4_ are 32.5% and 40.3%, respectively. Compared to the fresh 3Pt/MgFe_2_O_4_ and 3FeO_x_-3Pt/MgFe_2_O_4_, the spent 3Pt/MgFe_2_O_4_ and 3FeO_x_-3Pt/MgFe_2_O_4_ exhibits a higher content of adsorbed oxygen species. This indicates that the gaseous molecular oxygen could adsorb on the surface of the catalysts [33].

**Figure 7:**
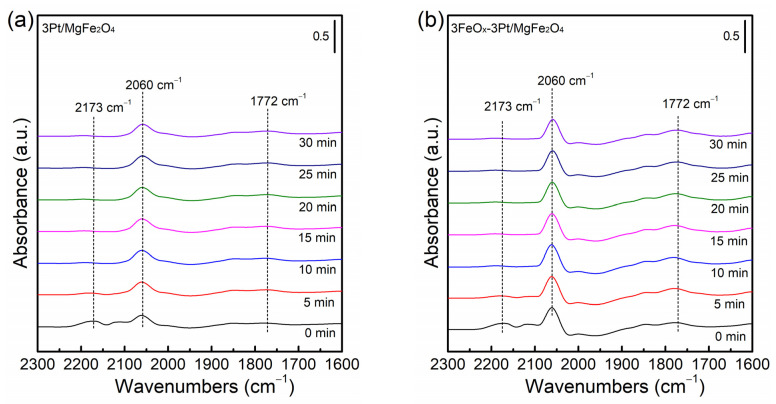
CO-DRIFT spectra of fresh (**a**) 3Pt/MgFe_2_O_4_ and (**b**) 3FeO_x_-3Pt/MgFe_2_O_4_.

Figure 7 shows CO-DRIFT spectra of fresh 3Pt/MgFe_2_O_4_ and 3FeO_x_-3Pt/MgFe_2_O_4_. Both 3Pt/MgFe_2_O_4_ and 3FeO_x_-3Pt/MgFe_2_O_4_ show bands at 2173, 2060 and 1772 cm^−1^, respectively. The band observed at 2173 cm^−1^ usually belongs to gaseous CO. The observed bands at 2060 and 1772 cm^−1^ are assigned to the linear and bridged CO adsorption on the surface of Pt^0^ [33,34,35,36,37], respectively. The findings suggest that the Pt species are primarily present in the form of a Pt^0^ metallic state in the pre-reduced catalysts. It is obvious that the CO bands’ intensities at 2060 and 1772 cm^−1^ on the 3FeO_x_-3Pt/MgFe_2_O_4_ catalyst are much higher than those on the 3Pt/MgFe_2_O_4_, indicating that the 3FeO_x_-3Pt/MgFe_2_O_4_ has a greater amount of adsorbed CO. 3FeO_x_-3Pt/MgFe_2_O_4_ with a high CO adsorption ability at 30 °C might result in an excellent CO-oxidation activity at low temperatures.

**Figure 8:**
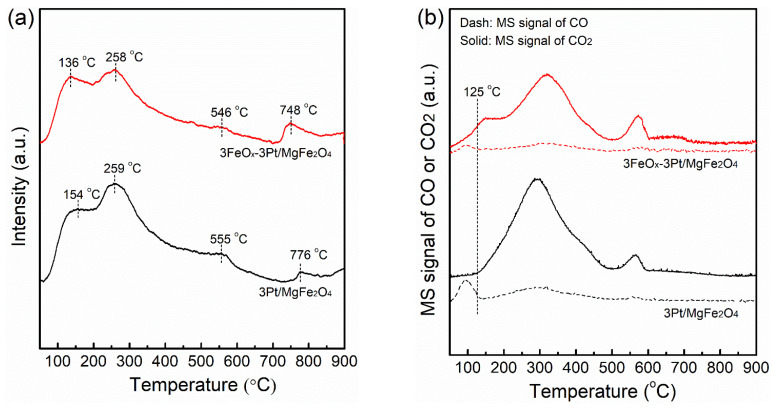
(**a**) O_2_-TPD and (**b**) CO-TPD profiles of the fresh 3Pt/MgFe_2_O_4_ and 3FeO_x_-3Pt/MgFe_2_O_4_.

Figure 8a shows the O_2_-TPD profiles of fresh 3Pt/MgFe_2_O_4_ and 3FeO_x_-3Pt/MgFe_2_O_4_. The O_2_-TPD profiles of 3Pt/MgFe_2_O_4_ show oxygen desorption peaks at 154, 259, 555 and 776 °C, respectively. These desorption peaks shifted to lower temperatures of 136, 258, 546, and 748 °C for 3FeO_x_-3Pt/MgFe_2_O_4_, respectively. The lower oxygen desorption temperature in 3FeO_x_-3Pt/MgFe_2_O_4_ confirmed that 3FeO_x_-3Pt/MgFe_2_O_4_ has a higher oxygen mobility, which is advantageous to catalytic-CO oxidation. Figure 8b presents the CO-TPD profiles of fresh 3Pt/MgFe_2_O_4_ and 3FeO_x_-3Pt/MgFe_2_O_4_. Both 3Pt/MgFe_2_O_4_ and 3FeO_x_-3Pt/MgFe_2_O_4_ exhibited small desorption peaks of CO and large desorption peaks of CO_2_. The formation of CO_2_ may be related to the surface reaction of the adsorbed CO species with oxygen species. It has been observed that CO_2_ formation in 3Pt/MgFe_2_O_4_ begins at 125 °C, while for 3FeO_x_-3Pt/MgFe_2_O_4_, CO_2_ formation occurs at 50 °C or even lower temperatures. The lower temperature at which CO_2_ begins to form demonstrates that the oxygen species could be easily released and could participate in CO oxidation. Therefore, CO is much easier to be oxidized over 3FeO_x_-3Pt/MgFe_2_O_4_.

## 3. Discussion

The introduction of FeO_x_ (3 wt.%) into 3Pt/MgFe_2_O_4_ can greatly enhance its low-temperature catalytic activity and stability in CO oxidation. CO can be fully oxidized to CO_2_ at temperatures as low as 30 °C over 3FeO_x_-3Pt/MgFe_2_O_4_ at WHSV = 30,000 mL/(g·h) (Figure 1a). At 30 °C, the CO-turnover rate on 3FeO_x_-3Pt/MgFe_2_O_4_ is calculated to be 0.024 mol/(mol_Pt_-s), which is higher than the C-turnover rates reported in the literature (0.002 mol/(mol_Pt_-s)) calculated at 50 °C on Pt/TiO_2_ with high Pt oxidation state and a Pt size of less than 1 nm [38]. In addition, according to the literature reports, the CO-turnover rates at 30 °C on Pt/Nb_2_O_5_ modified with FeO_x_ with a Pt size of 3.5 nm is calculated to be 0.012 mol/(mol_Pt_-s) [39], which is also lower than the CO-turnover rates on 3FeO_x_-3Pt/MgFe_2_O_4_. These results indicate that the FeO_x_-modified ultrafine Pt nanoparticles (~1 nm) prepared in our article exhibit excellent low-temperature activity for the oxidation of CO. In addition, during the 35 min duration test for stability at 60 °C, the 3FeO_x_-3Pt/MgFe_2_O_4_ exhibited a high and constant CO conversion of 100.0% (Figure 1b). The nitrogen physical adsorption results indicated that the FeO_x_ modification might slightly decrease the surface area of 3Pt/MgFe_2_O_4_ (Table 1). According to the XRD and STEM results (Figure 3 and Figure 4, Table 1), the ultrafine Pt particles (~1 nm) are highly dispersed on the MgFe_2_O_4_ support for 3Pt/MgFe_2_O_4_ and 3FeO_x_-3Pt/MgFe_2_O_4_, and FeO_x_ modification has little/no effect on the size of Pt. Based on the H_2_-TPR- and XPS-characterization results (Figure 5 and Figure 6), there is an interaction between FeO_x_ and Pt with an electron transfer from Pt to FeO_x_. Moreover, the addition of FeO_x_ increased the ratio of the adsorbed oxygen species and decreased the oxidizing extent of the metallic Pt. The latter resulted in an increase in Pt^0^ percentage in an oxidizing atmosphere. As we know, the Pt catalyst is present in an oxidizing atmosphere during a CO-oxidation reaction. The reduced Pt can be oxidized in this reaction mixture. It is generally believed that metallic Pt is more active in CO-oxidation reactions than in that of oxidized Pt [40,41]. The CO-DRIFT results demonstrated that the FeO_x_ modification increased the amount of CO adsorption on 3Pt/MgFe_2_O_4_ (Figure 7). The results of O_2_-TPD and CO-TPD showed that FeO_x_ promoted the oxygen mobility of 3Pt/MgFe_2_O_4_ and CO_2_ formation at low temperatures (Figure 8a,b). The high oxygen mobility in 3FeO_x_-3Pt/MgFe_2_O_4_ makes CO more easily oxidized, resulting in an outstanding catalytic performance for CO oxidation. The above results indicated that the incorporation of FeO_x_ into Pt/MgFe_2_O_4_ has successfully modified the electronic properties of Pt nanoparticles, while having almost no effect on the geometric structure of Pt nanoparticles. The relatively strong antioxidant capacity, the high proportion of adsorbed oxygen species, and a high amount of adsorbed CO as well as the good oxygen mobility of 3FeO_x_-3Pt/MgFe_2_O_4_ contributed to its high low-temperature activity and improved stability for CO oxidation.

## 4. Materials and Methods

### 4.1. Catalyst Preparation

A solid-phase method was employed to synthesize the MgFe_2_O_4_ support. In brief, 0.015 M of MgSO_4_ (Zesheng, Anqing, China), 0.03 M of Fe(NO_3_)_3_·9H_2_O (Guoyao, Shanghai, China), 0.12 M of NaOH (Guoyao, Shanghai, China) and 0.15 M of NaCl (Annaiji, Shanghai, China) were simultaneously poured into a mortar and grinded evenly for 40 min. The obtained mixture was subjected to calcination at 700 °C for 1 h with a ramp rate of 2 °C/min. Subsequently, it was washed with deionized water and dried at 120 °C for 12 h.

The Pt/MgFe_2_O_4_ with Pt loading of 3 wt.% was synthesized through the incipient wetness impregnation of MgFe_2_O_4_ support with H_2_PtCl_6_·6H_2_O (Damas-beta, Shanghai, China) dissolved in a certain amount of deionized water. After impregnation, the sample was subjected to drying at 60 °C under ambient air for 12 h and calcination at 500 °C in air for 5 h. After undergoing a repeated washing process with deionized water, the obtained solid was subjected to drying at 120 °C for 12 h and then reduction at 150 °C for 30 min in 5% H_2_/N_2_. Catalysts thus prepared were denoted as fresh 3Pt/MgFe_2_O_4_.

FeO_x_-modified Pt/MgFe_2_O_4_ with Fe loading of 3 wt.% (excluding the Fe content in MgFe_2_O_4_ support) was prepared by further incipient wetness impregnation of calcined 3Pt/MgFe_2_O_4_ with the appropriate amount of Fe(NO_3_)_3_·9H_2_O (Guoyao, Shanghai, China) dissolved in a certain amount of deionized water. After impregnation, the sample was treated in the same way as the 3Pt/MgFe_2_O_4_, obtaining the fresh 3FeO_x_-3Pt/MgFe_2_O_4_ catalyst. The catalysts after the stability test of 60 °C were denoted as the spent 3Pt/MgFe_2_O_4_ and 3FeO_x_-3Pt/MgFe_2_O_4_.

### 4.2. Catalyst Characterization

We conducted the surface-area testing of the as-prepared samples on a Micromeritics ASAP 2460 model adsorption instrument (Norcross, GA, USA). The Bruauer–Emmett–Teller (BET) method was employed with nitrogen as an adsorbate.

The content of Pt in the as-prepared samples was measured using inductively coupled plasma optical emission spectrometry (ICP-OES) with an Agilent 5110 instrument (Santa Clara, CA, USA).

We obtained X-ray diffraction (XRD) patterns of the as-prepared samples using a Rigaku SmartLab SE apparatus (Tokyo, Japan). The Cu Kα radiation with a wavelength of 0.15406 nm was utilized as the excitation source.

A JEOL JEM-2100F instrument (Tokyo, Japan) was employed to acquire the scanning transmission electron microscopy (STEM) images.

A Micromeritics AutoChem II 2920 instrument (Norcross, GA, USA) was utilized for performing the temperature-programmed reduction of H_2_ (H_2_-TPR). The sample (~0.1 g) was introduced into a quartz tube and maintained at 300 °C for 30 min under an Ar atmosphere. Subsequently, the sample was exposed to a 10% H_2_/Ar and the temperature was gradually elevated to 800 °C using a ramp rate of 10 °C/min.

X-ray photoelectron spectroscopy (XPS) was determined by employing an Al Kα X-ray on a Thermo Fisher ESCALAB 250 Xi apparatus (Waltham, MA, USA). A reference of the C 1s peak at 284.6 eV was employed to correct all the obtained binding energies.

A Bruker Tensor 27 spectrometer (Ettlingen, Germany) was employed to record the in situ CO-diffuse reflectance infrared Fourier transform (CO-DRIFT) spectroscopy. The sample was subjected to reduction in a H_2_ atmosphere at 150 °C for a duration of 30 min before CO adsorption. After cooling down to 30 °C under Ar, 20% CO/Ar of 30 mL/min was passed through the sample until the saturated adsorption of CO was reached. The 20% CO/Ar was subsequently switched to Ar and the data was collected.

The Micromeritics AutoChem II 2920 apparatus (Norcross, GA, USA) was utilized to conduct temperature-programmed desorption of O_2_ (O_2_-TPD). The sample (~0.1 g) was firstly subjected to reduction in a 10% H_2_/Ar atmosphere at 150 °C for a duration of 30 min. After cooling down to 60 °C under Ar, 3% O_2_/He was introduced for a duration of 1 h to achieve the saturated adsorption of O_2_. Finally, the sample was subjected to a heating process up to 900 °C in the presence of an Ar atmosphere, and the data was recorded.

The Microtrac Belcat II apparatus (Osaka, Japan) was utilized to conduct temperature-programmed desorption (TPD) of CO. A 0.1 g sample was subjected to prereduction in a 10% H_2_/Ar atmosphere at 150 °C for a duration of 30 min. After the temperature decreased to 50 °C in the presence of an Ar atmosphere, 10% CO/He was introduced for 1 h for the saturated adsorption of CO. Finally, the sample was subjected to heating up to 900 °C in Ar, and the mass signals corresponding to CO with *m*/*z* of 28 and CO_2_ with *m*/*z* of 44 were recorded.

### 4.3. Catalytic Performance Tests

The oxidation of CO was conducted at atmospheric pressure in a U-shaped quartz fixed-bed reactor. Prior to the catalytic testing, all samples were exposed to 5% H_2_/N_2_ of 150 °C for a duration of 30 min for reduction. For each activity and stability test, a mixture consisting of 50 mg of catalysts and 1g of quartz sand was utilized. The reactant mixture consisted of 1% CO, 1% O_2_ and 98% N_2_. The total flow rate was 25 mL/min, which corresponded to a weighted hourly space velocity (WHSV) of 30,000 mL/(g·h). An FGA 10 model infrared gas analyzer was employed to monitor online the concentrations of reactants and products. We calculated the CO conversion as (C_CO, inlet_ − C_CO, outlet_)/C_CO, inlet_ × 100%, in which C_CO, inlet_ and C_CO, outlet_ represent the inlet and outlet CO concentrations of the reactor, respectively.

## 5. Conclusions

To summarize, we successfully synthesized a 3FeO_x_-3Pt/MgFe_2_O_4_ catalyst with ultrafine Pt particles that had an average size of ~1 nm. The incorporation of FeO_x_ into the Pt/MgFe_2_O_4_ catalyst significantly boosted the low-temperature catalytic activity and stability of the Pt catalyst for CO oxidation. CO conversion could reach 100.0% on 3FeO_x_-3Pt/MgFe_2_O_4_ at temperatures as low as 30 °C, and the elevated stability was obtained with a CO conversion of 100.0% at 60 °C for a 35 min run under a WHSV of 30,000 mL/(g·h). It was found that modifying 3Pt/MgFe_2_O_4_ with FeO_x_ tunes the electronic properties while hardly changing the size of the Pt particles. Compared with 3Pt/MgFe_2_O_4_, the remarkably increased catalytic performance of 3FeO_x_-3Pt/MgFe_2_O_4_ for CO oxidation might be attributed to its relatively strong antioxidant capacity, high ratio of adsorbed oxygen species, a greater amount of adsorbed CO, and an increased oxygen mobility. Overall, a deeper understanding of the crucial structure of catalysts needed for oxidizing CO in this work will contribute significantly to the advancement of a more high-performance Pt-based catalyst for the oxidation of CO.

## Figures and Tables

**Table 1:** Properties of the 3Pt/MgFe_2_O_4_ and 3FeO_x_-3Pt/MgFe_2_O_4_.

Samples	S_BET_ (m^2^/g)	Pore Size ^a^ (nm)	Pore Volume (cm^3^/g)	Pt Loading (wt.%)	d_STEM_ (nm)	(Pt^0^) ^b^ %	(O_ads_) ^c^ %	(Pt^0^) ^d^ %	(O_ads_) ^e^ %
3Pt/MgFe_2_O_4_	93.8	11.3	0.29	3.1	1.2	43.1	31.7	24.5	32.5
3FeO_x_-3Pt/MgFe_2_O_4_	77.9	11.5	0.25	2.9	1.3	49.0	37.4	31.7	40.3

^a^ Average pore size calculated from BJH desorption branch. ^b^ Proportion of Pt^0^ in the fresh 3Pt/MgFe_2_O_4_ and 3FeO_x_-3Pt/MgFe_2_O_4_ determined by the ratio of peak area according to A_Pt0_/(A_Pt0_ + A_Pt2+_ + A_Pt4+_). ^c^ Proportion of O_ads_ in the fresh 3Pt/MgFe_2_O_4_ and 3FeO_x_-3Pt/MgFe_2_O_4_ determined by the ratio of peak area according to A_Oads_/(A_Olatt_ + A_Oads_). ^d^ Proportion of Pt^0^ in the spent 3Pt/MgFe_2_O_4_ and 3FeO_x_-3Pt/MgFe_2_O_4_ determined by the ratio of peak area according to A_Pt0_/(A_Pt0_ + A_Pt2+_ + A_Pt4+_). ^e^ Proportion of O_ads_ in the spent 3Pt/MgFe_2_O_4_ and 3FeO_x_-3Pt/MgFe_2_O_4_ determined by the ratio of peak area according to A_Oads_/(A_Olatt_ + A_Oads_).

## Data Availability

Data are contained within the article.
